# Exposure, Cytotoxicity and Cellular Uptake of Silver (Ag) and Gold (Au) Nanoparticles in Human Bronchial Epithelial Cells During Nanoparticle Synthesis

**DOI:** 10.3390/nano16110687

**Published:** 2026-06-01

**Authors:** Mosima Letsoalo, Charlene Andraos, Masilu Masekameni, Mary Gulumian

**Affiliations:** 1 Occupational and Environmental Exposure and Health Division, School of Public Health, University of the Witwatersrand, Parktown, Johannesburg 2193, South Africa; successletsoalo@gmail.com; 2 Toxicology Department, National Institute for Occupational Health, National Health Laboratory Services, Johannesburg 2000, South Africa; charlenea@nioh.ac.za; 3 National Health Laboratory Services, Johannesburg 2000, South Africa; 4 Development Studies, School of Social Sciences, University of South Africa, Pretoria 0003, South Africa; 5 Water Research Group, Unit for Environmental Sciences and Management, North-West University, Private Bag X6001, Potchefstroom 2520, South Africa; mary.gulumian@nwu.ac.za

**Keywords:** nanoparticles, occupational exposure, cytotoxicity, BEAS-2B cells

## Abstract

Silver (Ag) and gold (Au) nanoparticles (NPs) are widely used in biomedicine, electronics, and catalysis, but their potential toxicity raises occupational health concerns. This study assessed the cytotoxicity and cellular interactions of Ag and Au NPs in human bronchial epithelial cells (BEAS-2B) using a standardized OECD three-tiered approach, alongside characterization of lung-deposited surface area (LDSA) concentrations during NP synthesis, which remained within ranges typically reported in occupational environments. Transmission electron microscopy revealed that AgNPs formed irregular clusters (~8.7 nm primary size, >30 nm aggregates), whereas AuNPs remained spherical (~13.4 nm). Real-time cytotoxicity analysis (xCELLigence) showed acute toxicity of AgNPs at 5 μg/cm^2^, while AuNPs exhibited no cytotoxic effects. Dark-field and 3D hyperspectral imaging demonstrated that some AgNPs were internalized by BEAS-2B cells, whereas AuNPs remained mostly on the cell surface, indicating that uptake alone does not determine cytotoxicity. The greater dissolution potential of AgNPs and possible release of Ag^+^ ions may contribute to the enhanced cytotoxic effects observed in comparison to AuNPs, as suggested in previous studies. Although oxidative stress, mitochondrial dysfunction, and related cellular mechanisms were not directly assessed in the present study, the findings demonstrate differential cellular responses following nanoparticle exposure under realistic occupational exposure conditions. These results contribute to understanding nanoparticle–cell interactions and support the need for further mechanistic investigations to inform safer nanomaterial use.

## 1. Introduction

Silver (Ag) and gold (Au) metallic nanoparticles (NPs) are utilized in various applications, such as in biomedicine, electronics, and catalysis sensing, due to their exceptional physio-chemical properties [1,2,3]. AgNPs and AuNPs are among the most extensively synthesized engineered nanomaterials (NMs) for their use in various fields [1]. Studies show that AgNPs negatively affect cell membranes; disrupt signaling pathways; disrupt the cell cycle; and cause mitochondrial dysfunction, oxidative stress, DNA damage, and apoptosis [1,4,5,6,7]. Additionally, exposure to AgNPs may lead to uptake, translocation, and most likely, biotransformation within the body [1]. Numerous studies on the toxicity of AgNPs attribute it entirely or in part to ionic silver that has been released or dissolved [1,8,9,10,11]. In contrast, various studies on AuNP toxicity provide inconsistent results, with some claiming no toxicity while others report adverse effects caused by AuNPs [1,12,13,14,15,16,17]. Furthermore, a consensus regarding AuNP toxicity is that their toxic effects cannot be conclusively defined, as the mechanism of their action depends on their physio-chemical properties (shape, size, charge, coating agents) but also on the targeted cells, tissues, and/or organisms as well as on the type of testing itself [1,18,19,20]. To rigorously evaluate and explain the effects of ENPs, each distinct NP type must be tested on different cell cultures using standardized and trustworthy assay protocols. While numerous studies have investigated the toxicological effects of AgNPs and AuNPs, most research has focused on controlled laboratory exposures rather than real-world occupational exposure scenarios.

Recent advances in exposure science suggest that metrics such as lung-deposited surface area (LDSA) and particle number concentration may provide more biologically relevant exposure indicators than traditional mass concentration [21,22,23,24,25]. The lack of specific occupational exposure limits (OELs) for ENMs makes it particularly challenging to understand and interpret information retrieved through workplace monitoring [21,26,27,28,29]. Additionally, instead of basing OELs for NMs and NPs on particle mass concentration, they could be based on other parameters, such as the LDSA and/or particle number concentration [21]. To further emphasize this point, Levin et al. [30] indicated that measurements of particle surface area can provide a potentially more biologically relevant metric for exposure and risk assessment. LDSA is often referred to as the fraction of the total airborne particle geometric surface area concentration that would be deposited in the human lung [30,31] and can be measured using real-time instrumentation. Following the use of real-time instrumentation, NPs may be collected on filters to characterize the NPs emitted subsequently [21]. Thus, evaluating exposure to airborne NPs should be based on a multimodal strategy that employs various measurement and assessment techniques. However, limited studies have attempted to integrate exposure monitoring data with toxicological assessment to understand better the potential health implications of NP exposure in occupational settings. Building on previous work by Masekameni et al. [32], which quantified occupational NP exposure using particle number and mass concentration metrics, the present study extends the exposure assessment by incorporating LDSA as a more physiologically relevant metric of potential respiratory dose. LDSA provides improved relevance for inhalation exposure assessment as it better reflects particle deposition in the human respiratory tract compared to conventional mass- or number-based metrics. Thus, this study adopted the OECD three-tiered approach to evaluate exposure to Ag and Au NPs synthesized in a controlled laboratory environment. During the synthesis of the Ag and Au NPs, the emissions were monitored using a combination of real-time monitoring instrumentation and a gravimetric sampling technique for particles collected, which were further used for toxicity assessment. By combining exposure monitoring using LDSA concentrations to characterize potential inhalation exposure, alongside using representative liquid-based synthesized NPs for in vitro assays and imaging techniques to assess cellular responses to representative NPs, this study seeks to provide insight into the potential biological interactions of NPs generated during synthesis processes.

## 2. Materials and Methods

### 2.1. Study Design and Location

**Figure 1:**
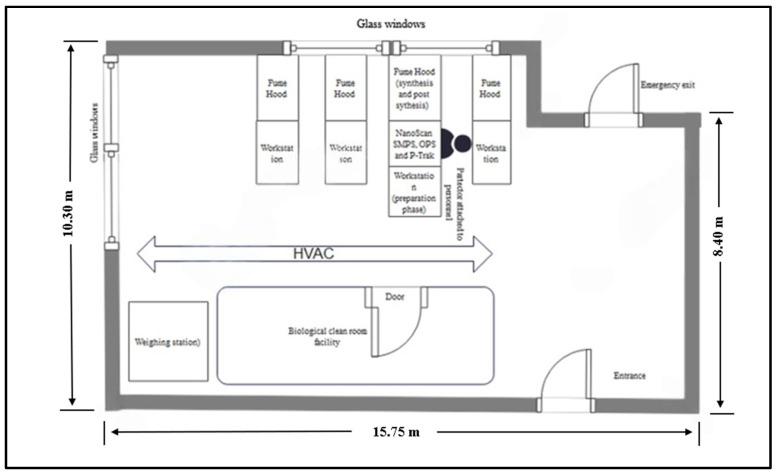
Schematic representation of the laboratory on the study site where the Ag and Au NPs were made. The figure is adapted from Masekameni et al. [32] due to the same experimental location and study environment being used in the current study.

The study adopted the OECD-tiered approach, where a walk-through was done for tier 1, encompassing laboratory layout, synthesis processes, and catalysts utilized. The standard operating procedure for the synthesis protocol was analyzed during the study. Tier 2 encompassed the use of real-time particle measurements to characterize potential inhalation exposure using LDSA concentration exposure dose metric during synthesis. During the synthesis of Ag and Au NPs, the LDSA concentration was measured using the partectorTEM (Naneos, Windisch, Switzerland) coupled with a 3 mm lacey carbon-coated 300 mesh copper grid. Moreover, the copper grids were analyzed with a Philips (FEI) CM100 TEM equipped with a Megaview III digital camera and coupled to an Oxford X-Max (80 mm^2^) to analyse the morphological characteristics. ImageJ 1.46r software was used to measure the diameter of individual NPs directly from the TEM images. Particle sizes were measured from multiple images to estimate the average particle diameter and morphological characteristics. Tier 3 involved toxicological evaluation using in vitro cell models to assess the potential biological effects of NPs associated with the identified exposure scenarios. Human bronchial epithelial cell lines (BEAS-2B) were exposed to the portion of NPs taken from a large quantity (bulk samples) of the synthesized NPs for toxicity assessment. Cytotoxicity assays were conducted using laboratory-prepared NP suspensions as representative model systems, rather than particles directly collected from ambient air. The collected particle mass was sufficient for TEM imaging and morphological analysis; however, the low particle mass yield was inadequate for reproducible in vitro cytotoxicity assays. A limitation of this study is that the particles used for cytotoxicity assessment were not directly sampled from the monitored environment, which may limit the direct translatability of the toxicological findings to real-world exposure scenarios. In addition, key physicochemical characterization parameters such as hydrodynamic size distribution, zeta potential, and dissolution behavior, which are known to influence nanoparticle toxicity, were not assessed in this study. Nevertheless, the absence of comprehensive physicochemical characterization represents a limitation that should be considered when interpreting the toxicity results. The study was conducted in a controlled laboratory environment, as shown in Figure 1, where the synthesis of Ag and Au NPs took place. The synthesis laboratory is located in Johannesburg, South Africa (26.1438° S, 27.9952° E).

### 2.2. Exposure Scenario

Both the Ag and Au NP measurements were conducted in the same laboratory on separate days. For Ag and Au NPs, the synthesis took place as described by Masekameni et al. [32]. As a result, exposure duration was calculated from the time spent on the synthesis steps. The worst-case scenario, accounting for variations in synthesis timeframes, involved two AgNP synthesis procedures and three AuNP procedures, resulting in an average exposure time of 360 min (6 h) over an 8 h work shift. The laboratory had a mechanical fresh air supply unit, no central heating, and ventilation, and the air conditioning (HVAC) system and ventilation was dependent only on the exhaust from the fume hood (0.93 m width × 0.75 m depth × 0.29 m sash opening height) and the fresh air supply. All synthesis and post-synthesis operations involving heating and chemical reactions took place under the fume hood. In contrast, preparation included weighing of precursor chemicals, measuring and transferring of the different reagents into appropriate vessels, and the placement of catalysts into reaction flasks, which according to the protocol, took place outside the fume hood. It was noted that there might have been a risk of exposure during the stirring phase and when taking the final product out of the fume hood. During the laboratory walkthrough, actual conditions (observations) are used to describe the exposure scenarios and laboratory layouts. Additionally, at the time of the investigation, the staff wore laboratory coats and disposable gloves as a typical procedure for working in a laboratory environment.

### 2.3. Toxicity Assessment

Toxic potencies (cell toxicity) of NPs were assessed with an xCELLigence real-time cell analyzer (RTCA) system version 2, as per manufacturer instructions. This served as an initial screening technique to determine the toxicity of NPs by observing their interaction with the cells. Thereafter, an Olympus BX43 microscope fitted with CytoViva dark-field hyperspectral imaging with a monochromatic camera was used to capture images from EZ slides to assess whether the NPs entered the BEAS-2B cells or merely remained on the surface of the BEAS-2B cells. In conventional bright-field microscopy, NPs are often difficult to visualize due to their small size and the limited optical contrast they produce relative to the surrounding cellular structures [33]. In contrast, dark-field microscopy enhances the visibility of NPs by illuminating the sample with oblique light while blocking directly transmitted light. As a result, only light scattered by the NPs reaches the objective, producing bright signals against a dark background and enabling improved visualization of NP–cell interactions. The CytoViva Hyperspectral Imaging System utilizes this dark-field principle, allowing enhanced detection of NPs within biological samples [34]. The images of the BEAS-2B cells with NPs captured from the EZ slides were at a 100X magnification.

#### 2.3.1. Preparation of Ag and Au NPs

The AgNPs and AuNPs stabilized with citrate were prepared by the Mintek research laboratory situated in Randburg, South Africa, as described by Masekameni et al. [32]. The final concentrations of AgNPs and AuNPs in Milli-Q water were 0.1 mg/mL.

#### 2.3.2. Cell Cultures

Similar to the study by Andraos et al. [16], the human bronchial epithelial cell lines were used in this study. The BEAS-2B cell line was obtained from Sigma Aldrich (catalogue number 95195102433) originally sourced from the European Collection of Cell Cultures, operated by the Health Protection Agency Culture Collections. This lung cell model was selected because (1) inhalation is thought to be the most likely exposure route for NP exposure [16,35,36,37] and (2) the BEAS-2B cell line is a common cell type for research on the nanosafety of inhaled NPs, especially for AgNPs and AuNPs [16,38].

#### 2.3.3. Seeding of Cells

Bronchial epithelial cells were grown in Roswell Park Memorial Institute (RPMI) 1640 growth media (Pan BioTech, Lonza, Switzerland) supplemented with 10% fetal bovine serum and 1% penicillin/streptomycin. The cell culture flask was kept in an incubator (Water Jacketed CO_2_ Incubator, Thermo Electron Corporation) in a humidified environment with 5% CO_2_ and a temperature of 37 °C. Culture medium was removed, and cells were briefly rinsed with 5ml of phosphate-buffered saline (PBS) solution (Capricorn Scientific: Catalogue Number PBS-1A) to eliminate residual serum. Trypsin (5 mL) was added for cell detachment, and then promptly removed to avoid overexposure. Cells were neutralized with RPMI-1640 medium, which was subsequently discarded before proceeding to seeding. The centrifuge phase occurred at 4 °C for 5 min at 1000 revolutions per minute (rpm). Thereafter, the supernatant was removed immediately to prevent the pellet from dissolving into the solution. Then, 3 mL of RPMI medium was added to resuspend the pellet of cells.

#### 2.3.4. Cell-Based Studies

The BEAS-2B cells were seeded in sterile 16-well E-plates with gold electrodes at the bottom (Costar Corning Inc.) at 5 × 10^4^ cells/cm^2^ in 100 µL cell culture medium (referred to as medium that contains RPMI, fetal bovine serum (FBS), and penicillin/streptomycin) per well. ENP exposure was conducted using a submerged in vitro exposure model, where ENPs were dispersed directly in the cell culture medium. Submerged exposure systems remain widely used in nanotoxicology for cytotoxicity screening and mechanistic toxicity assessment, particularly in bronchial epithelial cell models such as the BEAS-2B cell line [39,40]. This approach enables precise control of NP concentration, dispersion stability, and exposure duration, which is important for reproducible toxicity measurements. Furthermore, the cytotoxicity in this study was assessed using the xCELLigence RTCA system, which monitors real-time changes in cell adhesion and proliferation, making submerged exposure suitable for detecting NP-induced changes in cell viability. While air–liquid interface (ALI) systems can better mimic physiological inhalation exposure, previous studies have shown that ALI models do not always significantly improve the assessment of inflammatory or genotoxic responses compared with submerged in vitro exposure [41,42]. Therefore, submerged exposure remains an appropriate and widely applied method for initial cytotoxicity evaluation of engineered nanomaterials.

Thereafter, the cells were left to reach the exponential (log) phase after 24 h of seeding in an incubator (37 °C, 5% CO_2_). The cell culture medium was replaced with 100 µL cell culture medium containing NPs at different final concentrations as follows: AgNPs 0.1, 1,2, and 5 µg/cm^2^, and AuNPs 2.5, 5, 25, and 50 µg/cm^2^. The concentration ranges were based on commonly reported doses used in in vitro nanotoxicology studies involving bronchial epithelial cells, while also considering the higher biological reactivity generally reported for AgNPs compared with AuNPs. This concentration was informed by airborne NP exposure measurements collected during the synthesis process. Since occupational exposure monitoring provides airborne particle metrics such as LDSA concentrations, the in vitro doses were selected based on concentrations commonly reported in in vitro nanotoxicology studies involving bronchial epithelial cells. Moreover, free Ag^+^ ion release from AgNPs was not measured in this study; previous reports indicate that citrate-stabilized AgNPs can release measurable amounts of Ag^+^ under cell culture conditions. The cell plates had 16-well E-plates: 4 wells were used for controls, 4 were used for the AuNPs at different concentrations, and the other 4 were used for the AgNPs at different concentrations. The untreated cells used as controls had only 100 µL of cell culture medium without NPs added to the wells on the E-plates. Each cell exposure was conducted in triplicate per test.

### 2.4. Data Collection and Analysis

The study followed the OECD 3-tiered approach to gather data on synthesis processes, catalysts utilized, and laboratory layout. The LDSA concentrations were measured using the partectorTEM instrument.

#### 2.4.1. Exposure Monitoring

A partectorTEM sampler (Naneos, Switzerland) was attached to the waist belt of the laboratory personnel during the synthesis. Furthermore, PVC tubing was used to connect the partectorTEM, comprising a 3 mm lacy carbon-coated 300 mesh copper grid, to the partectorTEM probe on one end, and the other end was placed on the collar of the laboratory personnel’s coat. This was to enable the tubing to be within the circumference of the personnel’s breathing zone. This device measured the alveolar LDSA range of 0–20,000 µm^2^/cm^3^ and records time-resolved exposure data throughout the sampling period. The partectorTEM estimates LDSA concentration using an electrical diffusion charging method, which measures the surface area of airborne particles or agglomerates based on their charging behavior rather than their primary particle size. The partectorTEM’s manufacturer-provided interpretive ranges were used to contextualize the LDSA results: concentrations below approximately 50 µm^2^/cm^3^ are typically observed in low-particle environments, whereas concentrations exceeding 250 µm^2^/cm^3^ may indicate elevated NP exposure conditions. These values should be interpreted as practical reference ranges rather than regulatory exposure limits or toxicological thresholds. Salo et al. [22] assessed LDSA concentrations by comparing the obtained values to known ranges in ambient air (10–50 µm^2^/cm^3^), other occupational situations (about 59–220 µm^2^/cm^3^), and a prior study from a taconite mine that reported 54–303 µm^2^/cm^3^. Of note, LDSA measurements were utilized as a measure of potential respiratory exposure to airborne NPs during synthesis processes. While the LDSA metric provides an estimate of the particle surface area likely to deposit in the alveolar region of the lung, direct conversion of these measurements into equivalent in vitro doses remains challenging. Consequently, the toxicity experiments carried out in this study should be interpreted as indicative assessments of NP cytotoxicity rather than direct quantitative representations of occupational exposure levels.

In two different microcentrifuge tubes, 50 µL of AgNPs and AuNPs was added to the cell culture media, respectively. The solutions were added to the 3 mm lacy carbon-coated 300 mesh copper grids and allowed to dry for 10 min before analysis was done. Particles stuck to the TEM grid’s surface were photographed to determine their morphologies using the JEM-2100, a versatile 200 kV analytical electron microscope. The instrument is produced in Akishima, Tokyo, Japan, by JEOL Ltd. and is often used to study semi-structures, particularly in particles with a smaller diameter. For improved usability, the TEM microscope integrates the JEM-2100 optical system with a sophisticated control system. Dynamic Light Scattering (DLS) measurements were not performed in this study. The primary particle size and morphology of AgNPs and AuNPs were characterized using TEM, which allows for direct visualization of NP structure and size at the nanoscale. While DLS can provide information on hydrodynamic diameter in suspension, which can influence NPs’ toxicity, the emphasis of this study was placed on evaluating exposure scenarios and biological responses associated with NP synthesis processes.

#### 2.4.2. xCELLigence

Cytotoxicity was assessed using the xCELLigence Real-Time Cell Analysis (RTCA) system, which monitors cell proliferation, morphology, and viability in real time through impedance-based measurements. The AgNPs’ final concentrations in the cell culture medium were 0.1, 1,2, and 5 µg/cm^2^, while for AuNPs, final concentrations in the cell culture medium were at 2.5, 5, 25, and 50 µg/cm^2^. The dose in this study followed a similar pattern to that of Andraos et al. [16], where µg/cm^2^ represents the mass of NPs per unit surface area of the culture dish. It was reported that µg/cm^2^ was more relevant than µg/mL since most of the NPs tend to settle in the culture over time as a result of agglomeration and gravity [16]. Additionally, using µg/cm^2^ (based on the well surface area) in representing in vitro NPs enables accurate comparison with realistic in vivo conditions, i.e., µg of NP per surface area of the lung [16].

The 16-well E-plates were placed in the xCELLigence RTCA in an incubator. For 24 h, scans were obtained every 5 min; for the rest of the experiment, they were obtained every 15 min. To reduce inter-well variability and enable well comparison, the CI values were normalized at a specific time point, which was chosen as the moment immediately before the addition of NPs. Cell viability was assessed at 6, 12, 24, and 48 h following NP exposure, and statistical analysis was performed to determine whether significant differences in cell viability occurred between treated and control groups at each time point. The cytotoxicity endpoint for this study was the growth of cells/cell death. Thus, the endpoint was measured and translated into real-time Cell Index (CI) values, which were indicative of the level of adhesion associated with the viability of the cells. The CI values of treated cells were compared with those of untreated control cells to identify NP concentrations that produced statistically significant cytotoxic effects. The following equation was used to calculate CI, as adopted from Chung & Technologies [43]:

(1) $$Cell\;index\left(CI\right)=\frac{Rcell(fi)-Rb(fi)}{nominal\;impedance\;value\;[15\;\Omega \;(ohms)]}$$

where N is the number of frequency points at which the impedance is measured, R_cell(fi)_ is the frequency-dependent electrode impedance at any time, and Rb_(fi)_ is the background impedance measured at the initial time without cells.

#### 2.4.3. CytoViva

For CytoViva, the EZ slides (Merck) contained eight (8) wells. The first two (2) were the control, while the other four (4) contained AgNPs. The final concentration in the cell culture medium was 0.1 and 2 µg/cm^2^, while for AuNPs, the final concentrations were 2.5 and 25 µg/cm^2^. The imaging with CytoViva was conducted at 24 h and 48 h intervals.

The CytoViva hyperspectral imaging system was used at 100× magnification using the HSI System 1.1 and ENVI 4.8 software, and incorporated onto the CytoViva Unit to assess whether NPs were internalized within cells using CytoViva’s Spectral Angle Mapping (SAM) Software. During SAM, the software identified NPs based on their light-scattering properties, which are unique to each NP type. Additionally, the images were captured under 100× magnification using a Q Imaging EXi blue monochrome camera. The stacks were collected using a Z stage; thus, these stacks combine to form the 3D image. While individual Z-planes were not quantitatively analyzed, the Z-stack images allowed visualization of the spatial distribution of NPs within the cells, providing qualitative evidence suggestive of NP internalization. While this approach provides evidence of NP internalization, additional techniques such as TEM of cells or confocal microscopy would be required to fully confirm internalization.

### 2.5. Data Reliability and Validity

The manufacturer calibrated every instrument used in the study in accordance with the calibration specifications. Additionally, the instruments were calibrated both before and after monitoring, and any deviations were recorded. All key cell-based experiments, including cytotoxicity and imaging analyses, were performed using independent biological triplicates to ensure the reproducibility and robustness of the findings. Each experiment was independently repeated three times under identical experimental conditions. Data presented therefore reflects the combined results obtained from multiple independent experiments rather than a single experimental run. The mean values and standard deviations of the results are displayed. A *p*-value ≤ 0.05 was considered significant. All student T-tests were performed with Statistica 64 Version 10 (StatSoft Inc., Tulsa, OK, USA).

## 3. Results and Discussion

This study applied the OECD-tiered approach to evaluate occupational exposure during the laboratory synthesis of AgNPs and AuNPs. Real-time LDSA measurements were conducted during preparation, synthesis, and post-synthesis activities using the partectorTEM, with TEM analysis used for particle characterization. Exposure monitoring was performed under routine laboratory conditions, where synthesis activities occurred mainly inside a fume hood, while some preparation and handling activities took place outside the hood. The identified exposure conditions were subsequently integrated with in vitro toxicity assessment to evaluate potential biological effects associated with occupational NP exposure.

### 3.1. LDSA Concentration During Synthesis of AgNPs and AuNPs

The LDSA concentration measured across the entire AgNP synthesis process was 40.3 µm^2^/cm^3^, calculated from the time-resolved measurements recorded throughout the synthesis process, as shown in Table 1. Background LDSA measurements were conducted prior to the commencement of NP synthesis monitoring to establish baseline laboratory conditions. The preparation, synthesis, and post-synthesis phases were defined according to the duration of each phase, and the LDSA concentrations reported for each phase represent average values derived from continuous real-time monitoring over the entire phase duration. The measured LDSA concentrations during AgNP and AuNP synthesis were lower than concentrations reported in several occupational NP exposure scenarios reported in the literature. The relatively low concentrations observed in the present study may be attributed to the implementation of engineering control measures, including the use of a fume hood and general ventilation system during NP synthesis. Similar observations were reported by Iavicoli et al. [21], where LDSA concentrations ranging between 3.58 and 5.44 µm^2^/cm^3^ were measured in an NP synthesis laboratory preparing several types of nanoparticles in liquid solutions, with no substantial increase observed during synthesis activities.

The present findings nevertheless indicate the potential for airborne NP exposure during synthesis activities. Under similar experimental conditions to the current study, Masekameni et al. [32] applied the Multiple-Path Particle Dosimetry (MPPD) model and reported that emitted AgNPs and AuNPs, most of which were <100 nm, were predominantly deposited in the pulmonary region, followed by the tracheobronchial and head regions of the respiratory tract. Deposition within the lower respiratory tract may be of concern because some NPs can persist within biological systems and potentially contribute to adverse chronic health outcomes [32].

Higher LDSA concentrations have been reported in other occupational environments. Belosi et al. [44] measured LDSA concentrations ranging between 73 and 98 µm^2^/cm^3^ during an industrial spray-coating process involving AgNPs capped with quaternized hydroxyethyl cellulose under worst-case conditions. Similarly, Geiss et al. [45] reported elevated LDSA concentrations in occupational settings associated with NP-generating activities. Welding environments showed LDSA concentrations ranging from 24.7 to 761 µm^2^/cm^3^, with an average of 137 µm^2^/cm^3^, while non-occupational environments such as canteen kitchens generated concentrations ranging from 15 to 3927 µm^2^/cm^3^, with an average of 415 µm^2^/cm^3^ [45]. The comparatively higher LDSA concentrations observed during AuNP synthesis relative to AgNP synthesis in the current study may be related to differences in particle formation, size distribution, and airborne behavior.

Currently, there remains limited literature on exposure assessment during NP synthesis processes, particularly regarding LDSA measurements in laboratory environments [44]. This highlights the importance of the present study in contributing exposure-related data to an area where information remains limited. Although direct conversion between airborne LDSA and in vitro mass dose remains challenging, LDSA provides an estimate of the particle surface area likely to deposit within the alveolar region of the lung. Consequently, the toxicity findings reported in this study should be interpreted as indicative assessments of NP cytotoxicity rather than direct quantitative representations of occupational exposure levels.

**Figure 2:**
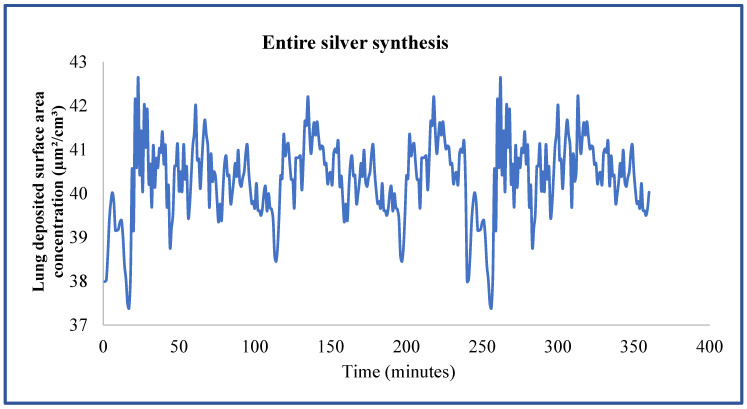
Time series LDSA concentration measured in the breathing zone during the synthesis of AgNPs using the partectorTEM.

As shown in Figure 2, the LDSA fluctuated throughout the entire synthesis process but remained below 43 µm^2^/cm^3^. Additionally, a stable LDSA concentration was noted that suggested a consistent synthesis process with minor variability due to fluctuations.

The synthesis of AuNPs resulted in an LDSA concentration of 78.6 µm^2^/cm^3^, as shown in Table 2. According to the interpretive ranges provided by the partectorTEM manufacturer, this concentration was above the lower exposure category threshold of 50 µm^2^/cm^3^ but remained below the higher exposure range of 250 µm^2^/cm^3^. These interpretive ranges should, however, be considered cautiously, as they do not represent regulatory occupational exposure limits.

**Figure 3:**
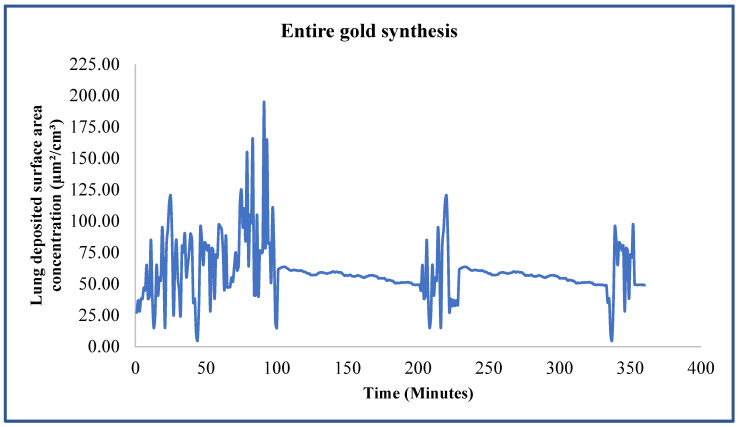
Time series LDSA concentration measured in the breathing zone during the synthesis of AuNPs using the partectorTEM.

A high variability of LDSA concentrations was noted, as shown in Figure 3, with peaks reaching as high as 200 µm^2^/cm^3^ before 100 min. Therefore, LDSA concentrations remained stable after 100 min, as shown in Figure 3. A spike was observed around 200 min, indicating that a temporary exposure event occurred.

### 3.2. Morphology of AgNPs and AuNPs

**Figure 4:**
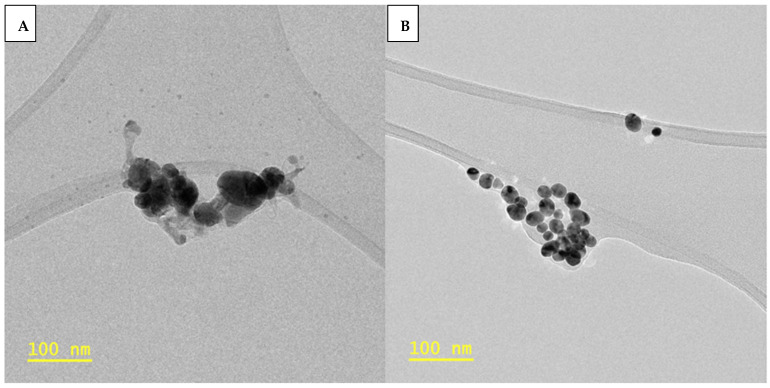
partectorTEM images of lacy carbon grids consisting of emitted (**A**) AgNPs and (**B**) AuNPs.

The morphology of NPs, such as size, shape, surface chemistry, and aggregation propensity, can influence their interaction with cells [46]. From Figure 4A,B, it is evident that the emitted AgNPs and AuNPs are spherically shaped.

**Figure 5:**
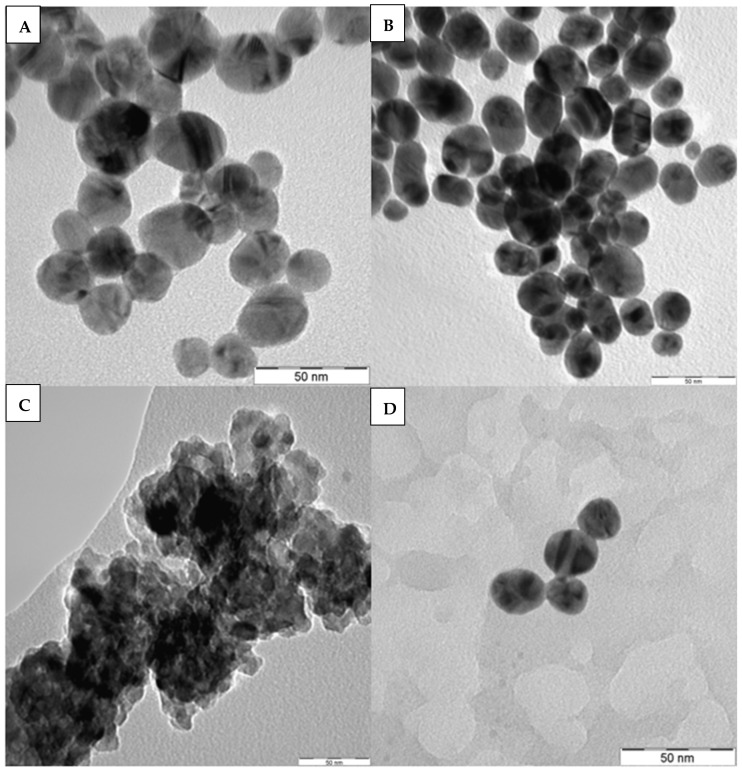
TEM images of lacy carbon grids consisting of (**A**) AgNPs in water solution, (**B**) AuNPs in water solution, (**C**) AgNPs in cell culture media, (**D**) AuNPs in cell culture media.

The TEM analysis in this study revealed that both AgNPs and AuNPs in a water solution (Figure 5A,B) primarily exhibited a spherical shape. Particle size was determined by measuring individual NPs directly from representative TEM micrographs using image analysis software. Measurements were performed across multiple randomly selected fields of view to ensure representativeness. The reported values reflect the mean ± standard deviation (SD) of measured particle diameters, with the size distributions appearing approximately unimodal. The average primary particle size of AgNPs in water was 20.84 ± 0.71 nm, while AuNPs exhibited a primary particle size of 19.2 ± 1.47 nm. In cell culture medium, the TEM analysis of AgNPs revealed the presence of irregular, aggregated structures. The measured particle size of individual AgNPs was 8.72 ± 1.95 nm; however, these primary particles were observed to form agglomerates ranging from tens to several hundreds of nanometers, as shown in Figure 5C. Thus, the smaller measured particle size reflects primary NPs, whereas the larger structures correspond to agglomerates formed in the biological medium. Agglomeration may influence the interpretation of uptake and toxicity results, as larger aggregates can alter sedimentation rates, diffusion behavior, and the effective dose delivered to cells. In addition, agglomerated structures may interact with cells differently compared to dispersed primary nanoparticles, potentially affecting uptake pathways and observed cytotoxic responses.

Similar to the study by Andraos et al. [16], the AgNPs in cell culture decreased slightly in particle size. This study showed a decrease in AgNPs in particle size. In a study by Kittler et al. [47], the interaction of bovine serum albumin (BSA) and fetal calf serum (FCS) with AgNPs resulted in the agglomeration of the AgNPs. AgNP agglomeration was observed to occur after 20 min of interaction with the biological media [47]. This finding is similar to that of the current study, as the TEM analysis of the AgNPs in cell culture media was done after 5 h of interaction. Furthermore, like in the current study, it was reported that AgNPs remain stable when exposed to RPMI for about 5 h, then rapidly agglomerate. This aggregation may influence NP uptake and biological responses. While DLS measurements would provide additional information on hydrodynamic particle size distribution in suspension, the current study focused on assessing exposure levels and cellular interactions using dark-field hyperspectral imaging (CytoViva) to confirm NP internalization. Physicochemical characterization parameters such as hydrodynamic size distribution, zeta potential, and dissolution behavior, which are known to influence NPs’ toxicity, were not assessed in this study. Consequently, the lack of comprehensive physicochemical characterization represents a limitation that should be considered when interpreting the toxicity results. Future studies should incorporate comprehensive physicochemical characterization of NPs in biological media to better understand their stability, behavior, and interactions under exposure conditions. For AuNPs in cell culture media, similar to the findings by Magogotya et al. [48], in this study, the AuNPs retained their spherical shape even after suspension in cell culture media, as seen in Figure 5D. AuNPs in cell culture media yielded a particle size of 13.40 ± 1.40, which was contrary to the findings in the study by Andraos et al. and Magogotya et al. [16,48], which reported an increase in particle size from AuNPs in water to AuNPs in cell culture media.

In the study by Iavicoli et al. [21], where colloidal suspension AgNPs were deposited on TEM grids, their analysis showed a size range of 4–5 nm. Furthermore, Iavicoli et al. [21] studied laboratory exposure to AuNPs, which, while differing in particle type and size from the present study, provided context on potential NP release during synthesis and the importance of monitoring occupational exposure in laboratory environments. Considering the current study, the size range of AgNPs in water was approximately 2–5 times larger (20.84 nm) than that reported by Iavicoli et al. [21]. In a study focusing on AgNP particle characterization in cell culture medium with added fetal bovine serum (CCM), alterations of the particle surface as a result of protein adsorption from the cell culture medium were reported [49,50]. Additionally, Hansen and Thünemann [49] reported that the concentration of AgNPs played a role, as a high concentration often resulted in a slower release of silver ions (Ag^+^) into the cell culture medium over time than lower concentrations of AgNPs. A study by Tomak et al. [51] using culture media containing 10% fetal bovine serum-supplemented Dulbecco’s Modified Eagle’s medium reported that TEM images demonstrated the adsorption of proteins, which led to an increase in the radius of AgNPs by 25 nm (from 50 nm to 75 nm) and agglomeration. A study by Maiorano et al. [50] reported a TEM observation of spherical morphology with no agglomeration for different-sized (15, 40, and 80 nm) AuNPs dispersed in ultrapure water. In addition, AuNP interaction with cell culture media achieved by suspending freshly synthesized batches of AuNPs in RPMI supplemented with 10% FBS yielded a size increase in the RPMI medium after 1 h [50]. AuNPs without and with cell culture medium (DMEM supplemented with 10% FBS, 100 mL penicillin, and 100 mg/mL streptomycin) and deposited onto a holey carbon-coated copper grid and dried for 24 h yielded high dispersion and an increase in AuNP size, i.e., 9.3 ± 0.6 nm to 101 ± 61 nm [52].

It has been reported that fetal calf serum (FCS) present in the culture medium can alter the surface charge of NPs, thus reducing their oxidative potential [53]. Additionally, Schlinkert et al. [53] elaborated that NPs may exhibit different properties in the presence of FCS than they would in the body.

### 3.3. Toxicity Assessment of BEAS-2B Cells Exposed to NPs

**Figure 6:**
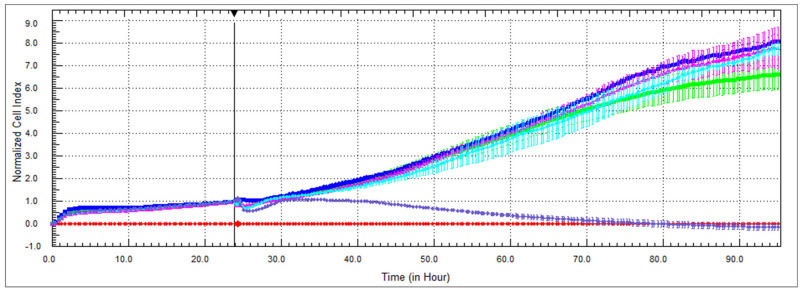
Normalized Cell Index of BEAS-2B cells seeded and allowed to recover for about 24 h before being treated with AgNPs at concentrations of 0.1 (dark blue), 1 (purple), 2 (light blue), and 5 (violet) μg/cm^2^. The CI curves were normalized (black vertical line) at 24 h for AgNPs.

**Figure 7:**
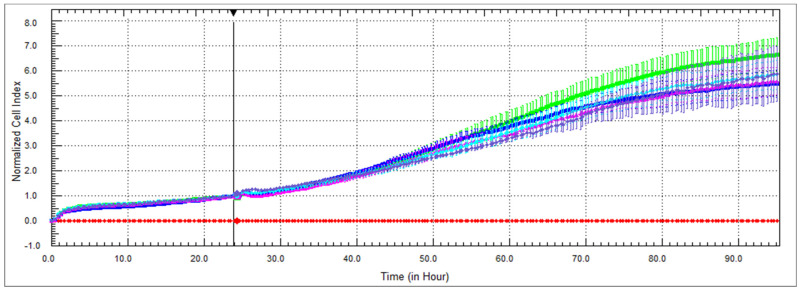
Normalized Cell Index of BEAS-2B cells seeded and allowed to recover for about 24 h before being treated with AuNPs at concentrations of 2.5 (dark blue), 5 (purple), 25 (light blue), and 50 (violet) μg/cm^2^. The CI curves were normalized (black vertical line) at 24 h for AuNPs.

For cell-free interference, Figure 6 and Figure 7 illustrate the growth curve for BEAS-2B cells treated with AgNPs and AuNPs, respectively, 24 h post-seeding using xCELLigence. Each line represents one treatment condition: control (untreated) and four NP concentrations. The increase observed in the negative control reflects normal cellular proliferation and attachment during the measurement period. In cell-based assays, untreated control cells typically show an increase in signal over time due to cell growth and metabolic activity. In contrast, treated cells may show reduced growth or altered response depending on the level of NP interaction or toxicity. Therefore, the upward trend observed in the control is consistent with normal cell behavior rather than experimental instability. In Figure 6**,** the acute toxicity of AgNPs against BEAS-2B cells was observed at 5 μg/cm^2^. The control cells exhibited a mean Cell Index of 2.258 ± 0.229. Exposure to AgNP concentrations of 0.1, 1, and 2 μg/cm^2^ resulted in mean Cell Index values of 2.64 ± 0.36, 2.68 ± 0.18, and 2.53 ± 0.19, respectively, with no statistically significant differences observed compared to the control group (*p* = 0.61, *p* = 0.40, and *p* = 0.64, respectively). In contrast, exposure to 5 μg/cm^2^ AgNPs resulted in a marked reduction in the Cell Index to 0.64 ± 0.07, which was statistically significant compared to the control (*p* < 0.001), indicating substantially greater cytotoxicity under this exposure condition.

Several studies have reported that AgNPs can release Ag^+^ ions in cell culture medium, and these ions are often partially responsible for the observed cytotoxic effects. Although we did not directly measure Ag^+^ release, the concentrations of AgNPs used and the cytotoxic responses observed in BEAS-2B cells are consistent with ranges reported in the literature, suggesting that both particle-mediated and ion-mediated mechanisms may contribute to the cellular effects. A limitation of the current study is that an ionic silver control was not included in the experimental design, which limits the ability to distinguish NP-specific effects from those potentially mediated by dissolved Ag^+^ ions. In addition, mechanistic endpoints such as reactive oxygen species (ROS) generation, mitochondrial dysfunction, inflammatory responses, apoptosis, NP dissolution, and dispersion stability were not directly evaluated. Physicochemical characterization parameters including hydrodynamic size, zeta potential, and agglomeration behavior in biological media were also not comprehensively assessed. Consequently, interpretations regarding the mechanisms underlying the observed cytotoxic effects remain hypothetical and are based primarily on findings reported in previous studies. Future work should incorporate ionic silver controls, detailed physicochemical characterization, dissolution studies, and mechanistic cellular assays to better understand NP behavior and toxicity under biologically relevant conditions.

A study by Muhamad et al. [54] using the MTT assay for BEAS-2B cells reported cell proliferation (cell growth) of over 50% at AgNP concentrations ranging from 12 to 35 µg/mL. Thus, at the reported concentrations, no toxicity was observed. Another study using the CCK-8 assay reported that at concentrations of 0.5, 0.75, 1, 1.25, and 1.5 µg/mL, the cell viability of BEAS-2B decreased to 77%, 33%, 19%, 11%, and 5%, respectively [55]. Additionally, the LD_50_ was approximately 0.65 µg/mL for 5 nm AgNPs, while particles > 100 nm did not exhibit any toxicity at concentrations up to 1.5 µg/mL [55]. Thus, Jang et al. [55] concluded that the toxicity of AgNPs was dependent on size and dose. A study by Gliga et al. [56] revealed that AgNPs of 10 nm showed cytotoxic effects on human lung epithelial BEAS-2B cells at concentrations of 20 and 50 μg/mL for 24 h, inhibiting mitochondrial activity and cell membrane integrity, while no cytotoxic effects were observed for 40 and 75 nm AgNPs when assessed by Alamar Blue (AB) and lactate dehydrogenase (LDH) assays. It has been reported that varying AgNP concentrations affect cell viability, immunological response, and bioaccumulation and cause double-strand DNA breakage, inflammation, and lung epithelium damage [37,46,56,57,58]. The study by Bachand et al. [59] highlighted that using WST-1 and IL-8 assays, exposure to AgNPs and AuNPs, whether at occupational and/or incidental levels, resulted in little to no acute cytotoxicity in alveolar epithelial cells. This was contrary to the current study’s findings, aligned with other studies, where the toxicity of the AgNPs on BEAS-2B was evident, in which a concentration of 5 μg/cm^2^ resulted in a decrease in cell viability. Researchers Andraos et al. [16] reported that at various concentrations (0.1, 0.5, 1,2, and 5 μg/cm^2^) of AgNPs, dose-dependent toxicity was observed with IC_50_ values calculated as 1.721 mg/cm^2^. This means that the toxicity of AgNPs was noted at a specific concentration. Although the occupational exposure measurements obtained in this study were expressed as LDSA concentrations (µm^2^/cm^3^), direct conversion between airborne LDSA and in vitro mass-based doses remains challenging due to differences in exposure metrics and deposition mechanisms. The observed cytotoxic effects are consistent with previous studies reporting reduced cell viability and proliferation in bronchial epithelial cells exposed to AgNPs at similar concentration ranges, supporting the biological relevance of the concentrations tested in the present study.

For AuNPs, the results indicate that although statistically significant differences were observed at certain AuNP concentrations, the magnitude of these changes was not indicative of acute cytotoxic effects under the conditions tested. Therefore, no acute cytotoxicity was concluded at the concentrations and time points evaluated in this study (Figure 7). Additionally, the absence of cytotoxicity in this experiment does not imply that AuNPs are inherently nontoxic. The control cells exhibited a mean Cell Index of 2.258 ± 0.229. Exposure to AuNPs at concentration 1 resulted in a mean Cell Index of 2.42 ± 0.164, which was not significantly different from the control group (*p* = 0.11). In comparison, AuNPs concentrations 2, 3, and 4 exhibited mean Cell Index values of 2.336 ± 0.163, 2.39 ± 0.204, and 2.35 ± 0.211, respectively, with statistically significant differences observed relative to the control group (*p* = 0.01, *p* = 0.05, and *p* = 0.019, respectively). Overall, the AuNP exposures resulted in relatively small changes in the Cell Index compared to the control cells. It has been reported that xCELLigence shows that AuNPs exhibit no toxicity against BEAS-2B cells even after 24 h [60]. Another study by Gambelunghe et al. [61] assessed the impact of AuNPs on BEAS-2B cell viability at different concentrations, namely, 0.8 and 1.6 µg/cm^2^, for a duration of 3, 24, and 48 h and concluded that the cells remained unaffected by the AuNPs at all tested concentrations and time points for the MTT assay. For BEAS-2B treated with 2.5, 5, 10, 25, and 50 μg/cm^2^ concentrations, xCELLigence revealed that AuNPs at these concentrations appeared to be nontoxic [16]. Like the above-mentioned studies, the current study showed that AuNPs at various concentrations did not result in changes in the BEAS-2B cell viability.

In this study, the observed cellular responses could not be directly attributed to the specific NPs measured in the occupational environment. Although LDSA provides a physiologically relevant indicator of potential respiratory deposition, translating this exposure metric into an equivalent in vitro dose remains challenging. A limitation of the current study is that bulk-synthesized NPs dispersed in liquid media were used for the in vitro experiments, whereas occupational exposure was characterized using aerosol measurements and TEM analysis. A direct quantitative comparison between the physicochemical properties of aerosol-collected NPs and the liquid-dispersed NPs used in the cytotoxicity assays was not performed. The aerosol-collected particles were primarily characterized by TEM to confirm the presence, morphology, and approximate size range of NPs generated during synthesis. Furthermore, the mass of particles collected during aerosol sampling was insufficient for repeatable cytotoxicity assessment.

Additionally, NP physicochemical properties may differ between airborne dry-state particles and liquid dispersions due to agglomeration, surface interactions, and dispersion behavior in biological media. However, the NPs used in the toxicity assays were synthesized using the same precursor materials, synthesis protocols, and laboratory conditions as those generating the occupational aerosols, thereby providing a representative indication of potential biological effects associated with workplace exposure. Future studies should incorporate air–liquid interface (ALI) exposure systems with direct aerosol deposition to better simulate realistic inhalation exposure conditions and improve the relevance of occupational NP risk assessment. Therefore, rather than being directly related, the exposure and toxicological results should be seen as complementary. To improve this relationship, future research should concentrate on combining aerosol collection with in vitro systems.

To contextualize the in vitro cytotoxicity doses relative to real-world exposure, lung-deposited doses were estimated from measured LDSA concentrations over an 8 h time-weighted average (TWA), incorporating body weight, inhalation rates (based on US EPA values), a deposition fraction of 0.4, and an assumed alveolar surface area of 75 m^2^. Based on these parameters, the estimated total deposited mass for AgNPs was approximately 6 mg for males (0.092 mg/kg) and 3.8 mg for females (0.045 mg/kg), while for AuNPs it was approximately 11–12 mg for males (0.11–0.12 mg/kg) and 8.3–9 mg for females (0.085–0.088 mg/kg). To enable comparison with in vitro conditions, these deposited doses can be approximated on a surface-area basis (µg/cm^2^) using the assumed alveolar surface area. These yields estimated exposure levels that are within or below the lower range of the administered in vitro doses (AgNPs: 0.1–5 µg/cm^2^; AuNPs: 2.5–50 µg/cm^2^). The higher in vitro doses were intentionally selected to evaluate dose–response relationships and potential hazards under elevated or worst-case exposure scenarios. It is important to note that this comparison represents a simplified approximation, as it does not account for factors such as particle clearance, agglomeration, or differences between aerosol-deposited and liquid-dispersed NPs. Therefore, while this approach provides a useful framework for contextualizing exposure relevance, it does not establish a direct equivalence between airborne LDSA measurements and in vitro dose metrics.

### 3.4. CytoViva

**Figure 8:**
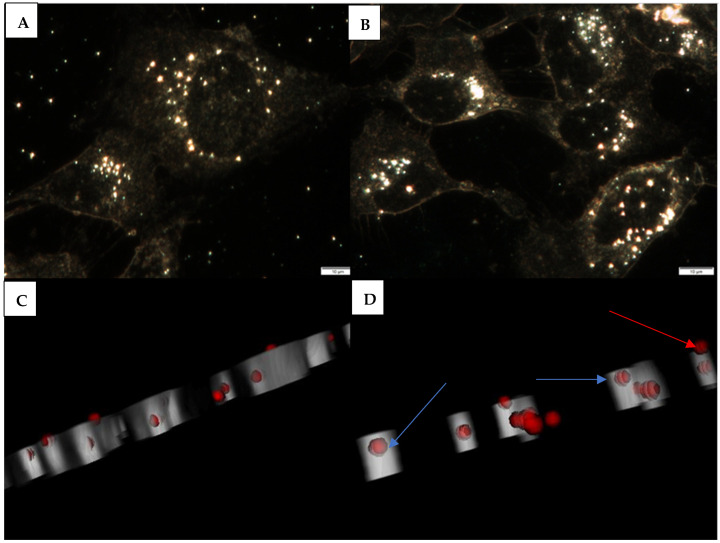
(**A**,**B**): Dark-field images acquired at 24 and 48 h, respectively, at 100× magnification, showing BEAS-2B cells treated with AgNPs. Interaction between AgNPs and BEAS-2B cells is illustrated in (**C**,**D**), which represent side-view 3D images captured at 24 and 48 h, respectively, at 100× magnification using a Q Imaging EXi blue monochrome camera. The blue arrows indicate AgNPs located inside the cells, while the red arrows indicate AgNPs attached to the cell surface.

**Figure 9:**
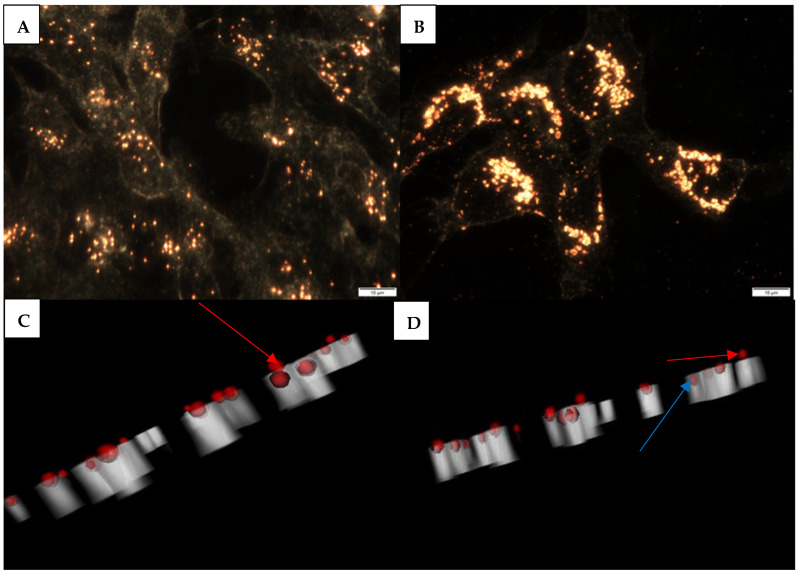
(**A**) and (**B**): Dark-field images acquired at 24 and 48 h, respectively, at 100× magnification, showing BEAS-2B cells treated with AuNPs. Interaction between AuNPs and BEAS-2B cells is illustrated in (**C**) and (**D**), which represent side-view 3D images captured at 24 and 48 h, respectively, at 100× magnification using a Q Imaging EXi blue monochrome camera. The blue arrows indicate AuNPs located inside the cells, while the red arrows indicate AuNPs attached to the cell surface.

For cell-free interference, the dark-field images of AgNPs and AuNPs exhibit bright and shiny points on a dark background, as shown in Figure 8 and Figure 9. Figure 8A,B show that AgNPs exhibit a blueish to greenish-white hue. In a study by Lu et al. [62], using CytoViva, it was observed that 10 nm AgNPs and AuNPs exhibited a greenish hue and a yellowish-brown hue, respectively.

The hyperspectral data cube acquisition provides three-dimensional data with two spatial (x, y) and one spectral (z) dimension [33,63]. When BEAS-2B cells were exposed to AgNPs, CytoViva imaging suggested that a substantial proportion of the NPs were associated with the cells and may have been internalized, as shown in Figure 8C,D. Real-time cytotoxicity monitoring with xCELLigence showed dose-dependent toxicity at 5 µg/cm^2^. These results are consistent with reports in the literature indicating that AgNPs are internalized into BEAS-2B cells via lysosomes and dispersed throughout the cytoplasm, nucleus, and mitochondria, as observed with fluorescence microscopy [54], or AgNPs are mostly found in the membrane-bound structures of BEAS-2B cells [56]. Internalization is likely to contribute to the cytotoxic effects observed.

AuNPs exhibit a brownish-orange hue, as shown in Figure 9A,B. Researchers Vetten and Gulumian [60] reported that no uptake of AuNPs in BEAS-2B cells was observed even after 24 h of exposure, whether at different concentrations using CytoViva. In the study by Vales et al. [64], the cellular uptake of AuNPs in BEAS-2B was assessed by hyperspectral imaging and yielded the internalization of AuNPs, especially clusters, in the cytoplasm but not in the nucleus. Like in other studies, AuNPs were predominantly located on the cell surface (Figure 9C,D). Although the 3D reconstructions provide suggestive evidence of internalization, further studies using TEM of cells or confocal imaging would be required for definitive confirmation. The limited uptake may be explained by particle size: 30 nm AuNPs show lower internalization efficiency compared to smaller particles (<20 nm) [65], consistent with previous findings in BEAS-2B cells [60,64]. Hyperspectral imaging using CytoViva indicated interaction and internalization of both AgNPs and AuNPs within BEAS-2B cells. However, qualitative observations suggested differences in NP localization. AgNPs appeared predominantly within the intracellular region, whereas a larger proportion of AuNPs were observed to be associated with the cell surface, with fewer particles detected within the cytoplasm. These observations suggest that both NP types are capable of interacting with, and potentially entering, cells, however the extent of intracellular accumulation may differ. Despite evidence of some cellular uptake for both NP types, only AgNP exposure resulted in a measurable reduction in cell viability. This suggests that particle internalization alone does not determine cytotoxicity. In the case of AgNPs, additional mechanisms such as NP dissolution and the release of Ag^+^ ions, induction of reactive oxygen species (ROS), oxidative stress, and mitochondrial dysfunction may contribute to the observed toxicological responses [56]. In contrast, AuNPs are generally considered more chemically inert and exhibit lower dissolution potential [66], which may explain the absence of significant cytotoxic effects despite cellular uptake. Therefore, the observed differences in cytotoxicity between AgNPs and AuNPs in the present study are likely influenced by differences in their physicochemical properties and ion release behavior rather than cellular uptake alone. These findings demonstrate that AgNPs and AuNPs exhibit distinct biological interactions and cytotoxic profiles, highlighting the importance of particle composition, size, and cellular uptake in assessing NP safety. While CytoViva imaging provided qualitative evidence of NP–cell interactions and intracellular localization, additional high-resolution techniques such as TEM of exposed cells would be required to definitively confirm intracellular localization and subcellular distribution. Additionally, the selection of an appropriate human lung epithelial cell model is essential for reliable in vitro toxicological studies [53].

An important aspect of this study was the integration of occupational exposure monitoring with in vitro toxicity assessment. LDSA measurements were used as an indicator of potential NP exposure during synthesis. Although LDSA is considered a biologically relevant exposure metric for airborne NPs, establishing a direct quantitative relationship between measured workplace exposure and in vitro dosing remains challenging. Consequently, the toxicity results presented here should be interpreted as indicative of potential biological responses rather than a direct representation of occupational exposure levels.

The lack of comprehensive physicochemical characterization of the NPs represents a limitation of the present study. Properties such as hydrodynamic size, surface charge (zeta potential), agglomeration behavior, and metal ion release can significantly influence NP toxicity and cellular interactions. In particular, the dissolution of AgNPs and subsequent release of Ag^+^ ions has been identified as an important contributor to cytotoxicity in many nanotoxicology studies. Future investigations should therefore include detailed physicochemical characterization to better understand the mechanisms underlying the observed biological effects.

Another limitation relates to the use of bulk-synthesized NPs rather than particles collected directly from the airborne exposure environment. While this approach allowed for a controlled laboratory evaluation of cytotoxicity, it may not fully represent the physicochemical characteristics of NPs present in occupational settings. Workplace-generated NPs can differ in morphology, size distribution, and state aggregation, which may influence their biological behavior.

Despite these limitations, the findings provide useful preliminary insights into the potential interactions between NPs and respiratory epithelial cells. The integration of exposure monitoring and toxicological assessment offers an important step toward improving the understanding of occupational risks associated with NP synthesis processes. Such combined approaches are increasingly recommended in nanosafety research to bridge the gap between exposure science and toxicological evaluation.

Future studies should aim to strengthen the linkage between workplace exposure measurements and biological response assessment by incorporating quantitative exposure–dose modeling, improved NP characterization, and additional mechanistic endpoints. Inclusion of oxidative stress markers, inflammatory responses, and advanced imaging techniques could further clarify the pathways involved in NP-induced cellular effects.

Overall, this study highlights the importance of evaluating both exposure and biological interactions when assessing the potential health implications of engineered nanomaterials. Continued research integrating workplace monitoring and toxicological testing will be essential for supporting risk assessment and developing appropriate occupational safety strategies for nanomaterial handling and production.

## 4. Conclusions

The measured LDSA concentrations during NP synthesis remained within ranges typically reported for occupational environments. CytoViva imaging demonstrated cellular internalization of AgNPs and, to a lesser extent, AuNPs in BEAS-2B cells; however, only AgNP exposure resulted in reduced cell viability at the highest tested concentration under the experimental conditions used in this study. This difference is likely related to the distinct physicochemical properties of the NPs, including the greater dissolution potential of AgNPs and the release of silver ions, which are known to contribute to cytotoxic responses.

Importantly, this work integrates workplace exposure characterization with in vitro toxicity assessment, contributing to efforts aimed at linking NP exposure with potential biological responses. While limitations such as incomplete physicochemical characterization and challenges in translating exposure metrics to in vitro doses should be considered, the results provide useful preliminary insights into NP–cell interactions relevant to occupational exposure scenarios.

Further studies incorporating detailed NP characterization and quantitative exposure–dose relationships will help strengthen the understanding of potential health risks associated with engineered nanomaterials.

## Figures and Tables

**Table 1:** Lung-deposited surface area concentration during the synthesis of AgNPs.

Phase	Lung-Deposited Surface Area Concentration (µm^2^/cm^3^)	Duration of Synthesis (Minutes)	Mode	Geometric Mean Diameter (GMD)	Geometric Standard Deviation (GSD)
Background	18.4	30	-	-	-
Preparation	40.2 ± 1.12	20	38.03	40.18	1.00
Synthesis	40.2 ± 1.13	42	40.86	40.19	1.03
Post-Synthesis	41.0 ± 0.51	94	40.91	40.98	1.01
Entire synthesis	40.3 ± 0.94	156	40.86	40.3	0.94

**Table 2:** Lung-deposited surface area concentration during the synthesis of AuNPs.

Phase	Lung-Deposited Surface Area Concentration (µm^2^/cm^3^)	Duration of Synthesis (Minutes)	Mode	Geometric Mean Diameter (GMD)	Geometric Standard Deviation (GSD)
Background	22.4	30	-	-	-
Preparation	55.61 ± 27.83	31	65	48.85	1.9
Synthesis	90.9 ± 67.54	36	75.0	73.0	3.0
Post-Synthesis	86.56 ± 41.77	64	65	76.03	2.1
Entire synthesis	78.6 ± 51.8	131	65	68.9	1.98

## Data Availability

All data analyzed during this study are included in this manuscript.

## References

[1] Pem B., Pongrac I.M., Ulm L., Pavičić I., Vrček V., Jurašin D.D., Ljubojević M., Krivohlavek A., Vrček I.V. (2019). Toxicity and safety study of silver and gold nanoparticles functionalized with cysteine and glutathione. Beilstein J. Nanotechnol..

[2] Abbas R., Luo J., Qi X., Naz A., Khan I.A., Liu H., Yu S., Wei J. (2024). Silver Nanoparticles: Synthesis, Structure, Properties and Applications. Nanomaterials.

[3] Burlec A., Corciova A., Boev M., Batir-Marin D., Mircea C., Cioanca O., Danila G., Danila M., Bucur A., Hancianu M. (2023). Current Overview of Metal Nanoparticles’ Synthesis, Characterization, and Biomedical Applications, with a Focus on Silver and Gold Nanoparticles. Pharmaceuticals.

[4] McShan D., Ray P.C., Yu H. (2014). Molecular toxicity mechanism of nanosilver. J. Food Drug Anal..

[5] More P.R., Pandit S., De Filippis A., Franci G., Mijakovic I., Galdiero M. (2023). Silver Nanoparticles: Bactericidal and Mechanistic Approach against Drug Resistant Pathogens. Microorganisms.

[6] Hermanto D., Ismillayli N., Fatwa D.H., Zuryati U.K., Muliasari H., Wirawan R., Prasetyoko D., Suprapto S. (2024). Bio-mediated electrochemically synthesis of silver nanoparticles using green tea (*Camellia sinensis*) leaves extract and their antibacterial activity. S. Afr. J. Chem. Eng..

[7] Kodintcev A.N. (2022). Characterization and potential applications of silver nanoparticles: An insight on different mechanisms. Chim. Technol. Acta.

[8] Wāng Y., Han Y., Xu D.X. (2024). Developmental impacts and toxicological hallmarks of silver nanoparticles across diverse biological models. Environ. Sci. Ecotechnol..

[9] Gao Y., Wu W., Qiao K., Feng J., Zhu L., Zhu X. (2021). Bioavailability and toxicity of silver nanoparticles: Determination based on toxicokinetic–toxicodynamic processes. Water Res..

[10] Jahan I., Bekler F.M., Tunç A., Güven K. (2024). The Effects of Silver Nanoparticles (AgNPs) on Thermophilic Bacteria: Antibacterial, Morphological, Physiological and Biochemical Investigations. Microorganisms.

[11] Zhang J., Wang F., Yalamarty S.S.K., Filipczak N., Jin Y., Li X. (2022). Nano Silver-Induced Toxicity and Associated Mechanisms. Int. J. Nanomed..

[12] Mateo D., Morales P., Ávalos A., Haza A.I. (2015). Comparative cytotoxicity evaluation of different size gold nanoparticles in human dermal fibroblasts. J. Exp. Nanosci..

[13] Shrivastava R., Kushwaha P., Bhutia Y.C., Flora S.J.S. (2016). Oxidative stress following exposure to silver and gold nanoparticles in mice. Toxicol. Ind. Health.

[14] Ramos-Pan L., Touzani A., Fernández-Bertólez N., Fraga S., Laffon B., Valdiglesias V. (2024). Impact of gold nanoparticle exposure on genetic material. Mutat. Res. Genet. Toxicol. Environ. Mutagen..

[15] Zhang L., Ma Y., Wei Z., Wang L. (2024). Toxicity of gold nanoparticles complicated by the co-existence multiscale plastics. Front. Microbiol..

[16] Andraos C., Yu I.J., Gulumian M. (2020). Interference: A Much-Neglected Aspect in High-Throughput Screening of Nanoparticles. Int. J. Toxicol..

[17] Vetten M.A., Tlotleng N., Rascher D.T., Skepu A., Keter F.K., Boodhia K., Koekemoer L.A., Andraos C., Tshikhudo R., Gulumian M. (2013). Label-free in vitro toxicity and uptake assessment of citrate stabilised gold nanoparticles in three cell lines. Part. Fibre Toxicol..

[18] Niżnik Ł., Noga M., Kobylarz D., Frydrych A., Krośniak A., Kapka-Skrzypczak L., Jurowski K., Nanoparticles G. (2024). Safety and Green Synthesis: A Critical Review. Int. J. Mol. Sci..

[19] Fratoddi I., Venditti I., Cametti C., Russo M.V. (2015). How toxic are gold nanoparticles? The state-of-the-art. Nano Res..

[20] Hu X., Zhang Y., Ding T., Liu J., Zhao H. (2020). Multifunctional Gold Nanoparticles: A Novel Nanomaterial for Various Medical Applications and Biological Activities. Front. Bioeng. Biotechnol..

[21] Iavicoli I., Fontana L., Pingue P., Todea A.M., Asbach C. (2018). Assessment of occupational exposure to engineered nanomaterials in research laboratories using personal monitors. Sci. Total Environ..

[22] Salo L., Rönkkö T., Saarikoski S., Teinilä K., Kuula J., Alanen J., Arffman A., Timonen H., Keskinen J. (2021). Concentrations and size distributions of particle lung-deposited surface area (LDSA) in an underground mine. Aerosol Air Qual. Res..

[23] Edebeli J., Spirig C., Fluck S., Fierz M., Anet J. (2023). Spatiotemporal Heterogeneity of Lung-Deposited Surface Area in Zurich Switzerland: Lung-Deposited Surface Area as a New Routine Metric for Ambient Particle Monitoring. Int. J. Public Health.

[24] Fung P.L., Zaidan M.A., Niemi J.V., Saukko E., Timonen H., Kousa A., Kuula J., Rönkkö T., Karppinen A., Tarkoma S. (2022). Input-adaptive linear mixed-effects model for estimating alveolar Lung Deposited Surface Area (LDSA) using multipollutant datasets. Atmos. Chem. Phys..

[25] Silvonen V., Salo L., Raunima T., Vojtisek-Lom M., Ondracek J., Topinka J., Schins R.P.F., Lepistö T., Lintusaari H., Saarikoski S. (2023). Lung-depositing surface area (LDSA) of particles in office spaces around Europe: Size distributions, I/O-ratios and infiltration. Build. Environ..

[26] Mihalache R., Verbeek J., Graczyk H., Murashov V., van Broekhuizen P. (2017). Occupational exposure limits for manufactured nanomaterials, a systematic review. Nanotoxicology.

[27] Gordon S.C., Butala J.H., Carter J.M., Elder A., Gordon T., Gray G., Sayre P.G., Schulte P.A., Tsai C.S., West J. (2014). Workshop report: Strategies for setting occupational exposure limits for engineered nanomaterials. Regul. Toxicol. Pharmacol..

[28] Schulte P.A., Kuempel E.D., Drew N.M. (2018). Characterizing risk assessments for the development of occupational exposure limits for engineered nanomaterials. Regul. Toxicol. Pharmacol..

[29] Visser M., Gosens I., Bard D., van Broekhuizen P., Janer G., Kuempel E., Riediker M., Vogel U., Dekkers S. (2022). Towards health-based nano reference values (HNRVs) for occupational exposure: Recommendations from an expert panel. NanoImpact.

[30] Levin M., Witschger O., Bau S., Jankowska E., Koponen I.K., Koivisto A.J., Clausen P.A., Jensen A., Mølhave K., Asbach C. (2016). Can we trust real time measurements of lung deposited surface area concentrations in dust from powder Nanomaterials?. Aerosol Air Qual. Res..

[31] Kuuluvainen H., Rönkkö T., Järvinen A., Saari S., Karjalainen P., Lähde T., Pirjola L., Niemi J.V., Hillamo R., Keskinen J. (2016). Lung deposited surface area size distributions of particulate matter in different urban areas. Atmos. Environ..

[32] Masekameni M.D., Andraos C., Yu I.J., Gulumian M. (2022). Exposure Assessment of Silver and Gold Nanoparticles Generated During the Synthesis Process in a South African Research Laboratory. Front. Toxicol..

[33] Zamora-Perez P., Tsoutsi D., Xu R., Rivera-Gil P. (2018). Hyperspectral-enhanced dark field microscopy for single and collective nanoparticle characterization in biological environments. Materials.

[34] Guttenberg M., Bezerra L., Neu-Baker N.M., Del Pilar Sosa Idelchik M., Elder A., Oberdörster G., Brenner S.A. (2016). Biodistribution of inhaled metal oxide nanoparticles mimicking occupational exposure: A preliminary investigation using enhanced darkfield microscopy. J. Biophotonics.

[35] De Berardis B., Marchetti M., Risuglia A., Ietto F., Fanizza C., Superti F. (2020). Exposure to airborne gold nanoparticles: A review of current toxicological data on the respiratory tract. J. Nanoparticle Res..

[36] Sonwani S., Madaan S., Arora J., Suryanarayan S., Rangra D., Mongia N., Vats T., Saxena P. (2021). Inhalation Exposure to Atmospheric Nanoparticles and Its Associated Impacts on Human Health: A Review. Front. Sustain. Cities.

[37] González-Vega J., García-Ramos J., Chavez-Santoscoy R., Castillo-Quiñones J., Arellano-Garcia M., Toledano-Magaña Y. (2022). Lung Models to Evaluate Silver Nanoparticles’ Toxicity and Their Impact on Human Health. Nanomaterials.

[38] Fortino V., Kinaret P.A.S., Fratello M., Serra A., Saarimäki L.A., Gallud A., Gupta G., Vales G., Correia M., Rasool O. (2022). Biomarkers of nanomaterials hazard from multi-layer data. Nat. Commun..

[39] Kim J.S., Peters T.M., O’Shaughnessy P.T., Adamcakova-Dodd A., Thorne P.S. (2013). Validation of an in vitro exposure system for toxicity assessment of air-delivered nanomaterials. Toxicol. Vitr..

[40] Lenz A.G., Karg E., Brendel E., Hinze-Heyn H., Maier K.L., Eickelberg O., Stoeger T., Schmid O. (2013). Inflammatory and oxidative stress responses of an alveolar epithelial cell line to airborne zinc oxide nanoparticles at the air-liquid interface: A comparison with conventional, submerged cell-culture conditions. BioMed Res. Int..

[41] Azqueta A., Stopper H., Zegura B., Dusinska M., Møller P. (2024). Pro-inflammatory and genotoxic responses by metal oxide nanomaterials in alveolar epithelial cells and macrophages in submerged condition and air-liquid interface: An in vitro-in vivo correlation study. Toxicol. Vitr..

[42] Jaber N., Billet S. (2024). How to use an in vitro approach to characterize the toxicity of airborne compounds. Toxicol. Vitr..

[43] Chung B., Technologies A. (2020). Using the xCELLigence RTCA Instruments to Perform Cell Adhesion Assays. https://www.agilent.com/cs/library/usermanuals/public/usermanuals-xcelligenct-rtca-single-plagte-5994-1894en-agilent.pdf.

[44] Belosi F., Koivisto A.J., Furxhi I., de Ipiña J.L., Nicosia A., Ravegnani F., Ortelli S., Zanoni I., Costa A. (2023). Critical aspects in occupational exposure assessment with different aerosol metrics in an industrial spray coating process. NanoImpact.

[45] Geiss O., Bianchi I., Barrero-Moreno J. (2016). Lung-deposited surface area concentration measurements in selected occupational and non-occupational environments. J. Aerosol Sci..

[46] Ahmed K.B.R., Nagy A.M., Brown R.P., Zhang Q., Malghan S.G., Goering P.L. (2017). Silver nanoparticles: Significance of physicochemical properties and assay interference on the interpretation of in vitro cytotoxicity studies. Toxicol. Vitr..

[47] Kittler S., Greulich C., Gebauer J.S., Diendorf J., Treuel L., Ruiz L., Gonzalez-Calbet J.M., Vallet-Regi M., Zellner R., Köller M. (2010). The influence of proteins on the dispersability and cell-biological activity of silver nanoparticles. J. Mater. Chem..

[48] Magogotya M., Vetten M., der Merwe M.P.R.-V., Badenhorst J., Gulumian M. (2022). In vitro toxicity and internalization of gold nanoparticles (AuNPs) in human epithelial colorectal adenocarcinoma (Caco-2) cells and the human skin keratinocyte (HaCaT) cells. Mutat. Res. Genet. Toxicol. Environ. Mutagen..

[49] Hansen U., Thünemann A.F. (2015). Characterization of Silver Nanoparticles in Cell Culture Medium Containing Fetal Bovine Serum. Langmuir.

[50] Maiorano G., Sabella S., Sorce B., Brunetti V., Malvindi M.A., Cingolani R., Pompa P.P. (2010). Effects of cell culture media on the dynamic formation of protein-nanoparticle complexes and influence on the cellular response. ACS Nano.

[51] Tomak A., Yilancioglu B., Winkler D., Karakus C.O. (2022). Protein corona formation on silver nanoparticles under different conditions. Colloids Surf. A Physicochem. Eng. Asp..

[52] López-Sanz S., Fariñas N.R., del C R., Ríos Á. (2019). Analytical strategy based on asymmetric flow field flow fractionation hyphenated to ICP-MS and complementary techniques to study gold nanoparticles transformations in cell culture medium. Anal. Chim. Acta.

[53] Schlinkert P., Casals E., Boyles M., Tischler U., Hornig E., Tran N., Zhao J., Himly M., Riediker M., Oostingh G.J. (2015). The oxidative potential of differently charged silver and gold nanoparticles on three human lung epithelial cell types. J. Nanobiotechnol..

[54] Muhamad M., Ab N., Omar W.A.W., Kamal N.N.S.N.M. (2022). Cytotoxicity and Genotoxicity of Biogenic Silver Nanoparticles in A549 and BEAS-2B Cell Lines. Bioinorg. Chem. Appl..

[55] Jang J., Park S., Choi I.H. (2021). Increased interleukin-11 and stress-related gene expression in human endothelial and bronchial epithelial cells exposed to silver nanoparticles. Biomolecules.

[56] Gliga A.R., Skoglund S., Wallinder I.O., Fadeel B., Karlsson H.L. (2014). Size-dependent cytotoxicity of silver nanoparticles in human lung cells: The role of cellular uptake, agglomeration and Ag release. Part. Fibre Toxicol..

[57] Kay J., Thadhani E., Samson L., Engelward B. (2019). Inflammation-induced DNA damage, mutations and cancer. DNA Repair..

[58] Ali N., Katsouli J., Marczylo E.L., Gant T.W., Wright S., de la Serna J.B. (2024). The potential impacts of micro-and-nano plastics on various organ systems in humans. EBioMedicine.

[59] Bachand G.D., Allen A., Bachand M., Achyuthan K.E., Seagrave J.C., Brozik S.M. (2012). Cytotoxicity and inflammation in human alveolar epithelial cells following exposure to occupational levels of gold and silver nanoparticles. J. Nanoparticle Res..

[60] Vetten G. (2019). Differences in uptake of 14 nm PEG-liganded gold nanoparticles into BEAS-2B cells is dependent on their functional groups. Toxicol. Appl. Pharmacol..

[61] Gambelunghe A., Giovagnoli S., Di Michele A., Boncompagni S., Dell’omo M., Leopold K., Iavicoli I., Talesa V.N., Antognelli C. (2020). Redox-sensitive glyoxalase 1 up-regulation is crucial for protecting human lung cells from gold nanoparticles toxicity. Antioxidants.

[62] Lu Q., Bhat A., Cooks T., Zhao J., Ren J. (2018). Dark-field microscopic study of the interactions between gold/silver nanoparticles and giant unilamellar vesicles. SPIE-Intl. Soc. Opt. Eng..

[63] Silva R.N., Botas A.M.P., Brandão D., Bastos V., Oliveira H., Debasu M.L., Ferreira R.A.S., Brites C.D.S., Carlos L.D. (2022). 3D sub-cellular localization of upconverting nanoparticles through hyperspectral microscopy. Phys. B Condens. Matter..

[64] Vales G., Suhonen S., Siivola K.M., Savolainen K.M., Catalán J., Norppa H. (2020). Size, surface functionalization, and genotoxicity of gold nanoparticles in vitro. Nanomaterials.

[65] Bromma K., Cicon L., Beckham W., Chithrani D.B. (2020). Gold nanoparticle mediated radiation response among key cell components of the tumour microenvironment for the advancement of cancer nanotechnology. Sci. Rep..

[66] Khlebtsov N., Dykmana L. (2011). Biodistribution and toxicity of engineered gold nanoparticles: A review of in vitro and in vivo studies. Chem. Soc. Rev..

